# Outcomes of Patients with Portal Vein Thrombosis Undergoing Live Donor Liver Transplantation

**Published:** 2014-05-01

**Authors:** R. F. Saidi, N. Jabbour, Y. Li, S. A. Shah

**Affiliations:** *Division of Organ Transplantation, Department of Surgery, Alpert Medical School of Brown University, Providence RI, USA*

**Keywords:** Live donor, liver transplantation, Portal vein thrombosis, Outcomes, Portal vein thrombosis, Prognosis

## Abstract

Background: Live donor liver transplantation (LDLT) for patients with portal vein thrombosis (PVT) creates several technical challenges due to severe pre-operative condition and extensive collaterals. Although deceased donor liver transplantation in patients with PVT is now routinely performed at most centers, the impact of PVT on LDLT outcomes is still controversial.

Objective: To determine the outcome of patients with PVT who underwent LDLT.

Methods: We reviewed the outcome of adult patients with PVT who underwent LDLT in the USA from 1998 to 2009.

Results: 68 (2.9%) of 2402 patients who underwent LDLT had PVT. Comparing patients with and without PVT who underwent LDLT, those with PVT were older (53 *vs *50 yrs), more likely to be male, had longer length of stay (25 *vs* 18 days) and higher retransplantation rate (19% *vs* 10.7%). The allograft and patient survival was lower in patients with PVT. In Cox regression analysis, PVT was associated with worse allograft survival (HR=1.7, 95% CI: 1.1–2.5, p<0.001) and patient survival (HR=1.6, 95% CI: 1.2–2.4, p<0.001) than patients without PVT.

Conclusions: Patients with PVT who underwent LDLT had a worse prognosis than those without PVT.

## INTRODUCTION

Portal vein thrombosis (PVT) is a common complication associated with cirrhosis; its prevalence is between 0.6% and 15% [1-5]. PVT used to be an absolute contraindication for liver transplantation. However, advances in surgical techniques and patient care make it possible to overcome PVT during liver transplantation. In the recent years, innovative surgical techniques such as thrombectomy, use of venous jump graft, and use of portal vein tributaries have been proposed to overcome the operative challenges. 

Live donor liver transplantation (LDLT) in patients with PVT has its own difficulties such as need for distal dissection of vascular pedicle of the hilum and restricted availability of a vein graft. The presence of PVT in the recipient has frequently been considered as a controversial issue for LDLT candidates. During surgery, the initial attempt to overcome PVT is thrombectomy, which is successful in the majority of cases. However, failed thrombectomy may necessitate vessel grafting. Vascular conduits can easily be obtained from the donor during the process of deceased donor liver transplantation. Therefore, the only kinds of vascular grafts left that can be utilized in LDLT are autologous, cryopreserved vessel graft, and prosthetic graft. Autologous vascular grafts are ideal because they carry no immunogenicity risk. 

In this report, we analyze the outcome of adult patients with PVT who underwent LDLT in the USA from 1998 to 2009.

## MATERIALS AND METHODS

We retrospectively queried the Scientific Registry of Transplant Recipients (SRTR) database for adult patients with PVT who underwent LDLT in the USA from 1998 to 2009. Patients who had liver retransplantation or pediatric LDLT were excluded from the study.

In the SRTR database, PVT status is reported at two different times; it is reported for liver transplant candidates (recorded as of the time of listing) and for transplant recipients (recorded as of the time of transplant). For analysis involving transplant recipients, the data of the latter field has been used. Occasionally, the PVT field in the candidate and recipient files did not correlate (2.0% of patients). We did not specifically make adjustments when the two PVT covariates were not in agreement. The data on pre-operative assessment of PVT was not available in the database. We collected the following information under recipient in both groups: patient age, sex, MELD score, hospital length of stay, waiting time prior to transplantation, recipient wait, recipient body mass index (BMI), rejection at one year, and retransplantation rate. The information collected on the donor included donor age, weight, BMI, and sex. Missing values were imputed with the mean values. 

Statistical analysis

χ^2^ and *Student’s t* tests were used for comparison of proportions and means, respectively. Graft and patient survival was the primary outcome measured. Kaplan-Meier survival analysis was used for allograft and patient survival estimates. Continuous variables were categorized using exploratory data analysis, and assumptions of proportional hazards were met by extended Cox regression models with time-dependent covariates. Variables with more that 20% missing values, were excluded from analyses. We originally included the following factors for unadjusted analysis: recipient age, sex, donor age and sex, diagnosis, MELD, length of stay, race, and acute rejection. An unadjusted comparison of survival was performed using the log-rank test. Hazard ratios (HR) were estimated using Cox proportional-hazards methodology and estimates. Multivariate Cox modeling was performed using potential risk factors and covariates that were found to be statistically significant in unadjusted Cox models. A p value <0.05 was considered statistically significant.

This study was reviewed by the University of Massachusetts Medical School Institutional Review Board (IRB) and deemed appropriate for exemption from IRB oversight as no personal identifiers were used among datasets. 

## RESULTS

There were 2402 adult patients who underwent LDLT from 1998 to 2009. The cohort, was then divided to those with (n=68) and without PVT (n=2334). The incidence of PVT among LDLT recipients was therefore 2.8% (95% CI: 2.2%–3.5%). Table 1 shows the characteristics of the two groups. The patients had a mean follow-up of 8.2 (range: 3–14) years. Comparing patients with and without PVT who underwent LDLT, those with PVT were older (53 *vs* 50 yrs), more likely to be male, had longer lengths of stay (25 *vs* 18 days) and higher retransplantation rate (19% *vs* 10.7%). The patient’s race and MELD score were comparable in both groups. Although cold ischemic time was longer in PVT group (3.6 *vs* 3.1 h), the difference was not significantly different.

**Table 1:** Characteristics of donor and recipients who underwent live donor liver transplantation, stratified by the presence of portal vein thrombosis

Variables	non-PVT (n=68)	PVT (n=2334)	p value
Recipient age	50.3	53	0.04
Recipient gender (male)	56.9%	69.1%	0.04
Recipient race			
White	91%	88%	0.4
Black	5%	8%	
Other	4%	4%	
Donor age	37.3	36.3	0.8
Donor gender (male)	57.2%	55.8%	0.9
CIT	3.1±5.9	3.6±6.0	0.2
LOS	17.8±20.5	24.6±29.6	0.006
MELD	14.6±5.7	14.6±4.7	0.6
Re-Tx	10.7%	19.1%	0.03

**Figure 1:**
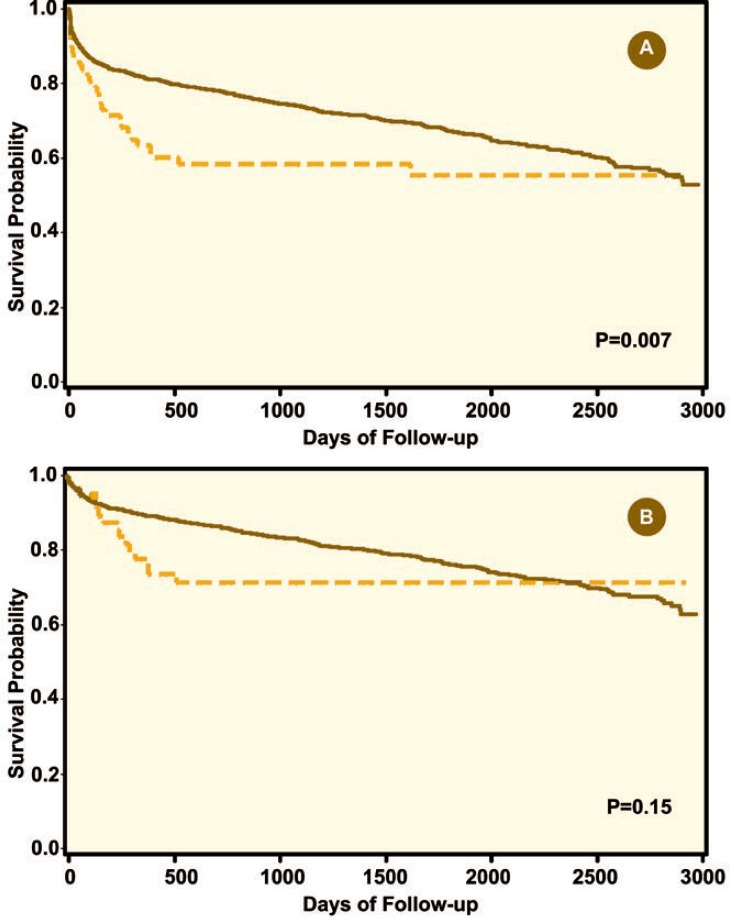
Allograft (A), and patients (B) survival for patients with portal vein thrombosis (dashed line) and without portal vein thrombosis (solid line) who underwent LDLT

**Figure 2:**
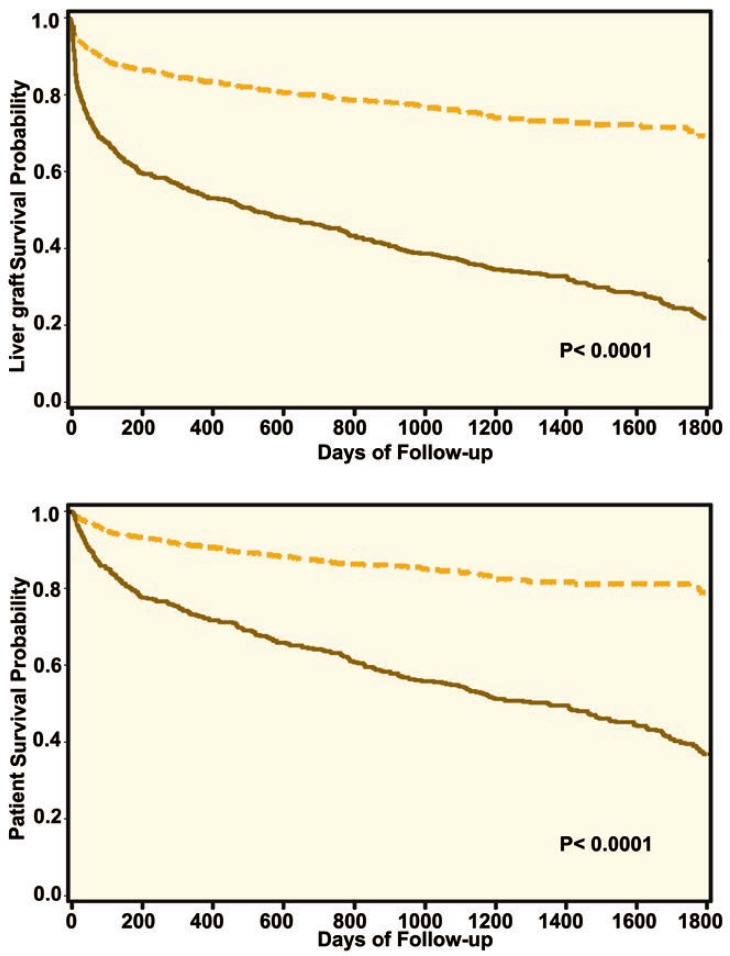
There was a significant improvement in results of LDLT for portal vein thrombosis comparing the first 34 cases (solid line) with the second 34 cases (dashed line) to address the learning curve issue

Overall, patients with PVT had worse allograft and patient survival when compared with those without PVT (Fig 1). In Cox regression analysis, PVT was associated with worse allograft survival (HR=1.7, 95% CI: 1.2–2.5, p<0.001) and patient survival (HR=1.6, 95% CI: 1.2–2.4, p<0.001) compared to non-PVT patients (Table 2). However, there was a marked improvement in the results of LDLT for PVT comparing the first 34 cases with the second 34 cases to address the learning curve issue (Fig 2). 

**Table 2A:** Cox proportional hazard model predicting allograft

Variables	HR (95% CI)	p value
Age	1.01 (1.01–1.03)	0.0028
Sex (female)	1.24 (101–1.54)	0.0380
PVT	1.7 (1.2–2.5)	<0.001
MELD	1.1 (0.89–1.23)	0.1
BMI	1.01(0.99–1.04)	0.07
Donor age	1.02 (1.01–1.03)	<0.001
Donor sex (female)	0.88 (0.71–1.07)	0.2

**Table 2B:** Cox proportional hazard model predicting patient survival

Variables	HR (95% CI)	p value
Age	1.01 (1.01–1.04)	<0.001
Sex (female)	1.23 (1.03–1.46)	0.01
PVT	1.6 (1.2–2.4)	<0.001
MELD	1.12 (0.98–1.45)	0.25
BMI	1.08 (0.95–1.1)	0.09
Donor age	1.0 (0.99–1.05)	0.06
Donor sex (female)	0.78 (0.68–1.11)	0.3

## DISCUSSION

Several groups have reported favorable results in patients with PVT who underwent liver transplantation, and have described effective strategies for the management of PVT during liver transplantation [1-5]. PVT is, therefore, no longer a contraindication for liver transplantation. 

LDLT has emerged as a solution to overcome the current organ shortage. The presence of PVT in the recipient has frequently been presented cautionary measure for LDLT by some groups based on the greater obstacles [6-8]. Pre-existing complete PVT creates considerable challenges during the surgery. The majority of centers consider PVT a relative contraindication for LDLT [8]. In this study, 2.8% of patients who underwent LDLT in the USA from 1998 to 2009 had PVT.

Another major concern in liver transplantation in patients with PVT is postoperative portal vein rethrombosis—6.2% to 28.6% of patients developed rethrombosis. Anticoagulation therapy to prevent portal vein rethromobosis after liver transplantation remains controversial, but it could be considered in high risk patients or for recanalization of PVT after liver transplantation.^7^ Taken together, depending on PVT grading and the experience of the surgeon, various surgical techniques can be performed during liver transplantation to restore adequate portal flow to liver allograft in patients with PVT,. Thrombectomy with direct portal vein anastomosis is the most commonly used operative approach [6-8]. Jump venous graft from the superior mesenteric vein is only indicated for the restoration of portal flow in cases of extensive PVT [8, 9]. Moreover, to ensure successful transplantation in patients with PVT, preoperative evaluation and thorough operative planning are essential. If PVT is extensive, re-establishment of portal flow needs complex vascular reconstruction. Specifically, in LDLT, the surgical techniques reported for the management of PVT include thrombectomy, jump graft to superior mesenteric vein or left renal vein [9], utilization of umbilical portion of recipient’s left portal vein [10], use of saphenous vein interposition graft [11], recipient’s explanted native right hepatic vein [12], and left gastric vein and iliac vein conduit from a deceased donor [13].

Previous single-center studies showed that the long-term outcome of patients with PVT who underwent LDLT, is comparable with that of patients without PVT [9, 15, 16]. Our study showed that patients with PVT who underwent LDLT had longer hospital stay and higher retransplantation rate. In addition, overall, allograft and patient survival was inferior comparing patients with and without PVT who underwent LDLT. PVT recipients were older, but had comparable MELD scores compared to non-PVT patients in this study. Our study showed that PVT is a risk factor for poor allograft and patient outcomes. Patients with PVT who underwent LDLT had early allograft lost and patient mortality (Fig 1), which could be due to post-operative complications or operative challenges. However, long-term survival can still be achieved in these patients. The outcome of patients with PVT who underwent LDLT has improved comparing the first 34 cases with the most recent 34 cases. This possibly can be explained with improvement in surgical technique, experience and peri- and post-operative care.

This study has several limitations. First, it is a retrospective analysis of SRTR data. We recognize both potential advantages and limitations of this study that use a large national database. However, the large sample size provides sufficient power to detect significant independent risk factors that may be missed by single-center studies. As with any analysis utilizing the SRTR database, our conclusions rely on the assumption that there is no systematic bias generated by reporting error or missing data. The groups were extremely unequal in size, selection criteria for one or the other procedures are not known. However, the primary endpoint for this analysis was allograft and patient survival, which is reliably captured in the SRTR database. Residual or unmeasured confounders that could impact allograft and patient survival include: surgeon technique, differences in immunosuppression protocols, the fat content/quality of the allograft and center-specific practices. Other important determinants of success with LDLT, such as recipient and donor selection, graft weight and quality, GWRW ratio, surgical details, techniques employed to treat PVT, degree of PVT (partial *vs* complete), center and surgeon volume/experience were not available in the database.

In conclusion, the US experience (1998–2009) showed that outcomes of patients with PVT who underwent LDLT were inferior to those without PVT. The lower allograft and patient survival should be taken into consideration in the overall debate regarding the choice of LDLT *vs* deceased donor liver transplantation in patients with PVT. LDLT should be performed under protocol and studied in experienced centers for patients with PVT.
